# Switching to Immune Checkpoint Inhibitors upon Response to Targeted Therapy; The Road to Long-Term Survival in Advanced Melanoma Patients with Highly Elevated Serum LDH?

**DOI:** 10.3390/cancers11121940

**Published:** 2019-12-04

**Authors:** Maartje G. Schouwenburg, Karijn P.M. Suijkerbuijk, Rutger H.T. Koornstra, Anouk Jochems, Michiel C.T. van Zeijl, Alfons J.M. van den Eertwegh, John B.A.G. Haanen, Maureen J.B. Aarts, Alexander C.J. van Akkooi, Franchette W.P.J. van den Berkmortel, Jan Willem B. de Groot, Geke A.P. Hospers, Ellen Kapiteijn, Wim H. Kruit, Djura Piersma, Rozemarijn S. van Rijn, Albert J. ten Tije, Gerard Vreugdenhil, Jacobus J.M. van der Hoeven, Michel W.J.M. Wouters

**Affiliations:** 1 Department of Medical Oncology, Leiden University Medical Centre, Albinusdreef 2, 2333 ZA Leiden, The Netherlands; A.Jochems@lumc.nl (A.J.); H.W.Kapiteijn@lumc.nl (E.K.);; 2 Dutch Institute for Clinical Auditing, Rijnsburgerweg 10, 2333 AA Leiden, the Netherlands; m.wouters@nki.nl; 3 Department of Medical Oncology, University Medical Centre Utrecht Cancer Center, Utrecht University, Heidelberglaan 100, 3584 CX Utrecht, The Netherlands; K.Suijkerbuijk@umcutrecht.nl; 4 Department of Medical Oncology, Radboud University Medical Centre, Geert Grooteplein Zuid 10, 6525 GA Nijmegen, The Netherlands; Rutger.Koornstra@radboudumc.nl; 5 Department of Medical Oncology, VU University Medical Centre, De Boelelaan 1118, 1081 HZ Amsterdam, The Netherlands; vandeneertwegh@amsterdamumc.nl; 6 Department of Medical Oncology, Netherlands Cancer Institute—Antoni van Leeuwenhoek hospital, Plesmanlaan 121, 1066 CX Amsterdam, The Netherlands; j.haanen@nki.nl; 7 Department of Medical Oncology, Maastricht University Medical Centre, P. Debyelaan 25, 6229 HX Maastricht, The Netherlands; mjb.essers.aarts@mumc.nl; 8 Department of Surgical Oncology, Netherlands Cancer Institute—Antoni van Leeuwenhoek hospital, Plesmanlaan 121, 1066 CX Amsterdam, The Netherlands; a.v.akkooi@nki.nl; 9 Department of Internal Medicine, Zuyderland Medical Centre Sittard, Dr. H. van der Hoffplein 1, 6162 BG Sittard-Geleen, The Netherlands; F.vandenberkmortel@zuyderland.nl; 10 Oncological Center Isala, Isala, Dokter van Heesweg 2, 8025 AB Zwolle, The Netherlands; j.w.b.de.groot@isala.nl; 11 Department of Medical Oncology, University Medical Centre Groningen, Hanzeplein 1, 9713 GZ Groningen, The Netherlands; g.a.p.hospers@umcg.nl; 12 Department of Medical Oncology, Erasmus MC Cancer Institute, ‘s-Gravendijkwal 230, 3015 CE Rotterdam, The Netherlands; qo5tc3@kpnmail.nl; 13 Department of Internal Medicine, Medisch Spectrum Twente, Koningsplein 1, 7512 KZ Enschede, The Netherlands; D.Piersma@mst.nl; 14 Department of Internal Medicine, Medical Centre Leeuwarden, Henri Dunantweg 2, 8934 AD Leeuwarden, The Netherlands; Rozemarijn.van.Rijn@ZNB.NL; 15 Department of Internal Medicine, Amphia Hospital, Molengracht 21, 4818 CK Breda, The Netherlands; atentije@amphia.nl; 16 Department of Internal Medicine, Maxima Medical Centre, De Run 4600, 5504 DB Eindhoven, The Netherlands; G.Vreugdenhil@mmc.nl

**Keywords:** melanoma, metastasis, targeted therapy, immune checkpoint inhibitors, lactate dehydrogenase, real-life data, prognostic factors

## Abstract

The prognosis of patients with advanced melanoma has improved dramatically. However, the clinical outcomes of patients with highly elevated serum lactate dehydrogenase (LDH) remain very poor. The aim of this study was to explore whether patients with normalized LDH after targeted therapy could benefit from subsequent treatment with immune checkpoint inhibitors (ICI). Data from all patients with BRAF-mutant metastatic melanoma with a highly elevated serum LDH at baseline (≥2× upper limit of normal) receiving first-line targeted therapy between 2012 and 2019 in the Netherlands were collected. Patients were stratified according to response status to targeted therapy and change in LDH at start of subsequent treatment with ICI. Differences in overall survival (OS) between the subgroups were compared using log-rank tests. After a median follow-up of 35.1 months, median OS of the total study population (*n* = 360) was 4.9 months (95% CI 4.4–5.4). Of all patients receiving subsequent treatment with ICI (*n* = 113), survival from start of subsequent treatment was significantly longer in patients who had normalized LDH and were still responding to targeted therapy compared to those with LDH that remained elevated (median OS 24.7 vs. 1.1 months). Our study suggests that introducing ICI upon response to targeted therapy with normalization of LDH could be an effective strategy in obtaining long-term survival in advanced melanoma patients with initial highly elevated serum LDH.

## 1. Introduction

Multiple effective systemic treatment options have emerged for patients with advanced BRAF-mutant melanoma over the last decade. Since the approval of the BRAF inhibitor vemurafenib [1] and the CTLA-4 antibody ipilimumab [2], combination therapy with a BRAF and MEK inhibitor [3] and treatment with anti-PD-1 antibodies as monotherapy [4,5] or combined with a CTLA-4 antibody [6] have broadened the therapeutic arsenal for these patients. Combination therapy with a BRAF and MEK inhibitor has resulted in a median overall survival of over two years [7], while treatment with anti-PD-1 also concurrently showed significant improvements, with 2-year survival rates of 55–58% [8]. Nevertheless, although long-term survival may be achieved in a subgroup of patients, there is still an unmet medical need for patients with unfavorable prognostic factors [7,9]. Elevated serum lactate dehydrogenase (LDH) level is a well-known marker for poor outcome, and a strong negative predictor for response to immune checkpoint inhibitors (ICI) and targeted therapy [7,8]. In previous reports, substantially less activity was demonstrated in patients with elevated serum LDH of ≥2× upper limit of normal (ULN), with a median OS of 2.9 months after ipilimumab therapy [9] and 2.3 months after anti-PD1 therapy [10]; compared to 14.7 months and 16.1 months for patients with normal LDH, respectively. Similarly, LDH has been shown to be one of the key predictors of survival for patients receiving targeted therapy [11]. Although the majority of BRAF mutant patients with elevated serum LDH respond to targeted therapy, responses are usually short-lived, with median progression-free survival shorter than six months for patients with LDH ≥2× ULN, compared to 17 months for the patients with normal LDH [7]. 

Targeted therapies are capable of inducing rapid anti-tumor responses associated with a decrease in LDH [7], which might enable ICI to work more efficiently in patients with initial elevated serum LDH. Furthermore, BRAF and MEK-inhibition could facilitate immune responses in multiple ways. Preclinical data showed an increase in CD8+ T-cell recognition of tumor cells by inducing rapid upregulation of MHC class I surface expression in BRAF-mutant melanoma cells [12,13]. These data support the potential of BRAF-inhibition to increase response rates to ICI. Although this concept seems promising, clinical data supporting the approach of BRAF inhibitor induction treatment preceding treatment with ICI in patients with aggressive disease are lacking, and little is known about which patients could benefit from induction treatment.

This prospective population-based study focuses on the clinical outcomes of BRAF-mutant metastatic melanoma patients with baseline serum LDH of ≥2× ULN treated with first-line targeted therapy. The main objective of the study was to investigate whether the level of LDH and response status at the switch to ICI was associated with survival.

## 2. Results

### 2.1. Overall Study Population

**Figure 1:**
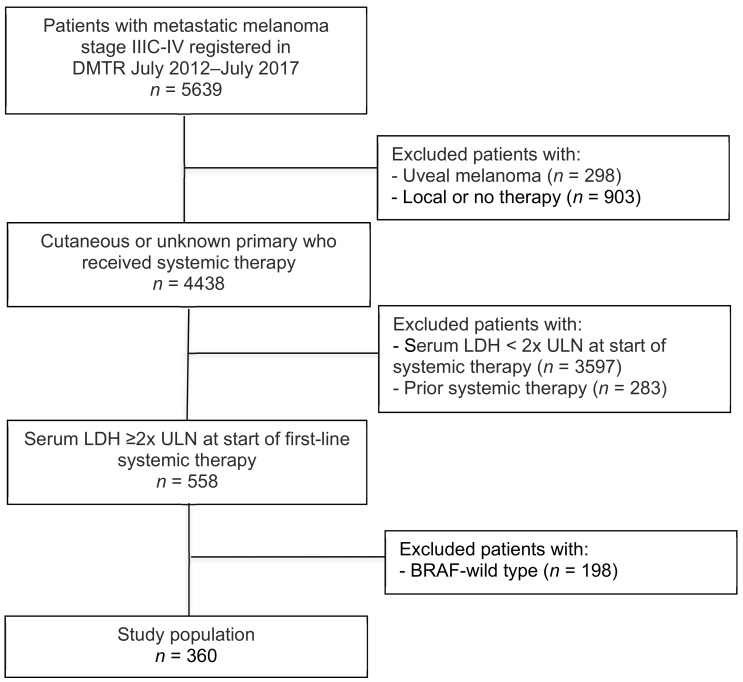
Flowchart of study population.

A total of 5639 unresectable stage IIIC or IV melanoma patients were registered in the Dutch Melanoma Treatment Registry (DMTR) between 1 July, 2012 and 1 June 1, 2019 (Figure 1). Of these, 360 BRAF-mutant advanced melanoma patients with a baseline serum LDH of ≥2× ULN received first-line targeted therapy and were included for analyses. Baseline characteristics are shown in Table 1. The median age was 60 years and the majority of patients were male (60%). Median serum LDH was 823 U/L (IQR 625–1419). Thirty eight percent of patients had an Eastern Cooperative Oncology Group performance status (ECOG PS) of ≥2 and most patients had ≥3 organ sites involved (72%). The majority of patients received BRAF monotherapy (76%). BRAF monotherapy was mainly prescribed up to August 2016. Combination therapy with a BRAF- and MEK inhibitor was increasingly used since October 2015. Median follow-up was 35.1 months (95% CI 18.2–52.1) and 308 patients (85%) died during follow-up. At time of analysis, 91% of patients discontinued treatment with targeted therapy, due to disease progression (60%), toxicity (10%), death (9%), planned in advance (11%), patient’s choice (2%), other (4%) and unknown (4%). 

**Figure 2:**
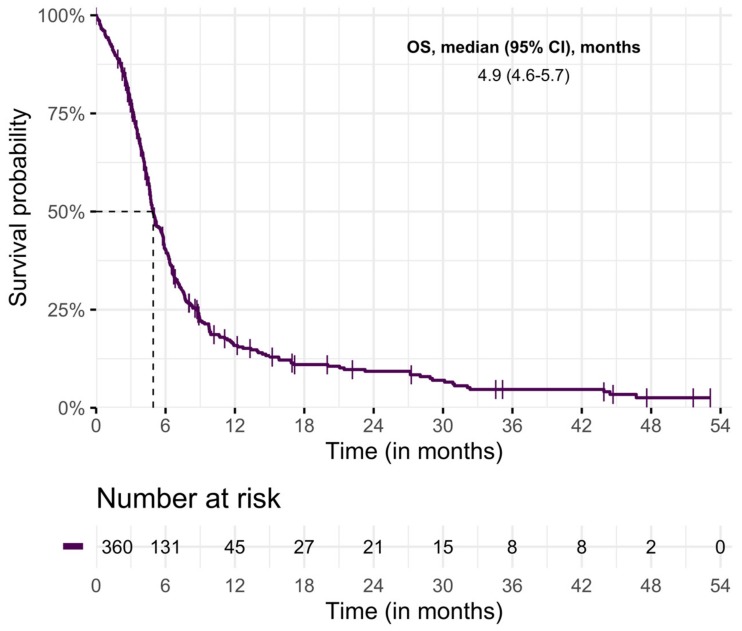
Overall survival of study population.

Median OS was 4.9 months (95% CI 4.4–5.4) (Figure 2). Survival rates at six months and one year were 40% (95% CI 35–45) and 16% (95% CI 12–20), respectively. 

### 2.2. Patients with Subsequent Treatment with ICI

A total of 113 patients (31%) received subsequent treatment with ICI. Combination therapy of ipilimumab and nivolumab was most often administered (*n* = 55), followed by anti-PD1 (pembrolizumab (*n* = 20), nivolumab (*n* = 16)), and ipilimumab (*n* = 22). Baseline characteristics at start of subsequent treatment with ICI are shown in Table 2. Median follow up from start of subsequent treatment with ICI was 30.0 months (95% CI 10.6–51.2).

The main objective of the study was to investigate whether the response to targeted therapy and level of serum LDH at start of subsequent treatment with ICI affects survival. Outcomes were stratified according to LDH at start of subsequent treatment with ICI and tumor response after targeted therapy. Table 3 shows the median OS and 6-months survival rates, calculated from start of subsequent treatment with ICI. 

Patients with a normalized LDH who had a partial response to prior targeted therapy (*n* = 16; combination therapy of BRAF and MEK inhibitor (*n* = 11), BRAF monotherapy (*n* = 5)) had the best survival from start of treatment with ICI (median OS 24.7 (95% CI 16.1–33.4) and 6-months and 1-year survival rate of 85% (95% CI 66–100) and 73% (95% CI 46–100), respectively). In this subgroup, most patients received combination therapy of ipilimumab and nivolumab (*n* = 9), followed by anti-PD1 (*n* = 6) and ipilimumab (*n* = 1). Median duration of targeted therapy before switching to ICI in LDH-normalized patients was 3.6 months (range 1.8–30.9). The main reason for treatment switch to ICI was a planned switch (*n* = 9). Other reasons were toxicity (*n* = 3) and unknown (*n* = 4). Baseline characteristics at start of targeted therapy were compared between the subgroup with normalized LDH and partial response, and the other subgroups. No significant differences were found (Table S1).

**Figure 3:**
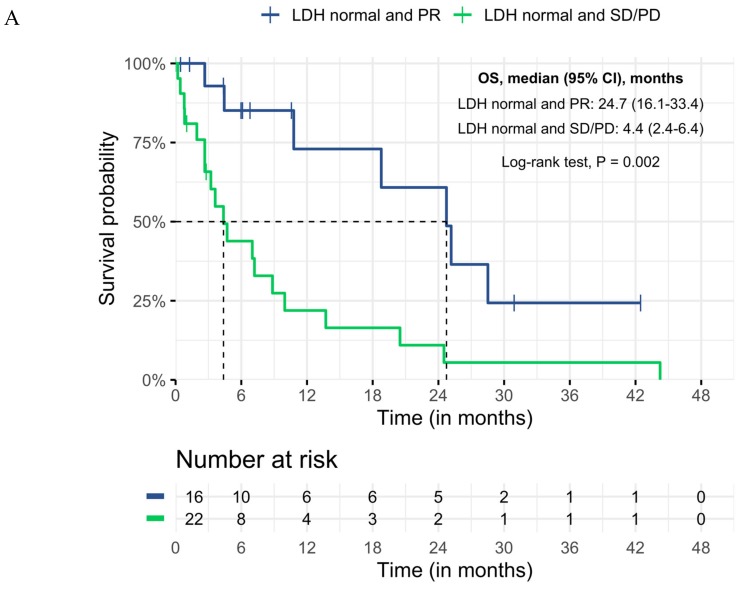

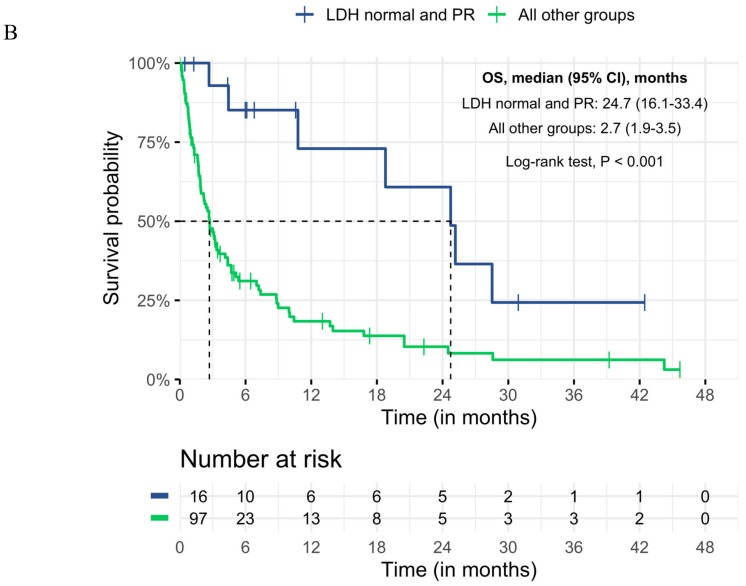
Differences in Kaplan–Meier curves of overall survival at start of subsequent treatment with ICI, in the subgroup with normalized LDH and PR compared to (**A**) normalized LDH and SD or PD, (**B**) all other subgroups, LDH = lactate dehydrogenase, OS = overall survival, CI = confidence interval, PR = partial response, SD = stable disease, PD = progressive disease.

Most patients who had an elevated LDH at start of treatment with ICI had progressed on targeted therapy (*n* = 63). Median duration of targeted therapy before switching to ICI was 5.9 months (95% CI 5.3–6.6). Patients who started second-line ICI with LDH ≥ 2× ULN had the worst outcomes, with a median OS of 1.1 months (95% CI 0.7–1.6), and 6-months and 1-year survival rate of 17% (95% CI 3–30) and 8% (95% CI 0–19), respectively. The survival curves demonstrate significant survival differences between the normalized LDH group with partial response, compared to the other subgroups (Figure 3a,b).

The 6-months and 1-year survival rates of the subgroup with normalized LDH and partial response are significantly better when compared to the whole subgroup that received ICI (6 months: 85% (95% CI 66–100) vs. 31% (95% CI 21–41); and 1-year: 73% (95% CI 46–100) vs. 18 (95% CI 10–27)).

## 3. Discussion

These real-world data support previous reports of the poor prognosis of advanced melanoma patients with highly elevated serum LDH. At the same time, these data provide a potential strategy to improve clinical outcomes. In our cohort of metastatic melanoma patients with baseline serum LDH of ≥2x ULN treated with first-line BRAF(/MEK) inhibitors, median OS was significantly longer in patients with normalized LDH and still responding to initial targeted therapy who started second-line treatment with ICI, compared to those with elevated LDH at start of treatment with ICI. Our data suggest that introducing ICI upon response to targeted therapy with normalization of LDH could be an effective strategy in obtaining long-term survival in patients with initial elevated serum LDH.

The median OS of 4.9 months of the overall study population confirms previous data, as clinical outcomes remain poor in this subgroup of patients [9,10,14]. Patients who received subsequent treatment with ICI with LDH levels that remained ≥2× ULN are unlikely to benefit from ICI with a median OS of 1.1 months and a 6-months and 1-year survival rate of 17% and 8%, respectively.

The exact role of LDH is not completely elucidated. It could simply be a marker of more aggressive disease that requires rapid anti-tumor responses [9]. The delayed tumor responses generally observed with ICI might therefore take too long for these patients to benefit. Moreover, tumor metabolism is characterized by the conversion of pyruvate into lactate, even in the presence of sufficient oxygen. Preclinical data demonstrated that tumor cells producing high levels of lactic acid disturb the function of cytotoxic T lymphocytes, thereby negatively influencing the potency of an immune response [15,16].

Interestingly, our data show that patients who switch to ICI with normalized LDH while still responding to targeted therapy have a real chance of long-term survival. After a median follow-up of 30 months, median OS was 24.7 months, and 6-months and 1-year survival rate was 85% and 73%, respectively. Survival was significantly longer compared to the other subgroups. No differences were found in prognostic factors at start of targeted therapy among the subgroups, indicating that this subgroup is not simply a selection of a best prognosis group.

It should be noted that only a small proportion of patients received this treatment strategy (*n* = 16; 4% of total study population). However, the majority of the total study population (*n* = 360) received BRAF monotherapy as first-line targeted therapy. The emergence of combination therapy with a BRAF and MEK inhibitor for this subgroup of patients might lead to a greater proportion of patients with response to targeted therapy and normalization of LDH. A 3-year follow-up pooled analysis of phase III trials with BRAF and MEK inhibitor combination therapy showed promising results, with 50% partial response in patients with initial LDH ≥ 2× ULN [17].

The value of sequencing targeted therapy prior to treatment with ICI in patients with initial elevated LDH has not been investigated thus far. Previous retrospective reports revealed that normalization of LDH while on targeted therapy was associated with ipilimumab cycle completion [18,19]. In another study on 101 advanced melanoma patients with decreased serum LDH after BRAF inhibitor treatment who completed all courses of ipilimumab, showed a significantly longer OS compared to those who did not (median OS 12.7 months vs. 1.2 months) [20].

The real benefit of induction treatment with combined BRAF- and MEK-inhibition in patients with elevated LDH is currently under investigation in multiple prospective randomized trials. The EORTC EBIN study (NCT03235245), compares ipilimumab and nivolumab upfront versus the same treatment preceded by induction therapy with encorafenib and binimetinib in advanced melanoma patients, irrespective of LDH level. One of the arms of the three-arm phase II SECOMBIT study (NCT02631447) will assess whether an induction treatment with encorafenib plus binimetinib of 8 weeks before combination therapy with ICI might help potentiate an immunotherapeutic response. Guidelines are not conclusive on this issue. Our results may therefore be of added value to medical oncologists while awaiting these trial results.

It would be interesting to investigate survival differences between patients who started second-line treatment with ICI with normalized LDH and response to initial targeted therapy vs. responders who stayed on targeted therapy. Unfortunately, this could not be assessed with our data, as we have no information on LDH level during follow-up with patients who stayed on targeted therapy.

Given the observational design of this analysis, we cannot rule out confounding by indication or selection bias. However, its multicentered design attenuates this potential selection bias. Furthermore, observational studies are more susceptible to registration bias. To ensure high-quality data, data managers were extensively trained and supervised by oncologists [21]. Another limitation is the small number of patients in the subgroup analyses. The conclusions drawn need validation in prospective randomized trials. Lastly, other clinical parameters such as lymphocyte counts and CRP level that have also been associated with patient outcome after treatment with ICI were not registered in our database, and could therefore not be included in this study [16].

## 4. Materials and Methods

### 4.1. Data: The Dutch Melanoma Treatment Registry (DMTR)

Data were retrieved from the Dutch Melanoma Treatment Registry (DMTR), a prospective population-based registry that was set-up to monitor the safety and effectiveness of the new drugs in real-world clinical practice and to assess the quality of melanoma care in the Netherlands. The DMTR contains information on baseline patient and tumor characteristics, local and systemic treatment modalities, treatment-related adverse events (grade 3 or 4 according to common terminology criteria for adverse events (CTCAE) version 4), and clinical outcomes of all patients with unresectable stage IIIC or IV melanoma. A detailed description of the DMTR was published previously [21].

In compliance with Dutch regulations, the DMTR was approved by the medical ethical committee and was not subject to the Medical Research Involving Human Subjects Act. Patients were offered an opt-out option.

### 4.2. Patients

All patients with BRAF-mutant unresectable or metastatic (stage IIIC or stage IV) cutaneous melanoma or with a BRAF-mutant melanoma of unknown primary with a baseline serum LDH of ≥2x the upper limit of normal (ULN), who received targeted therapy (either monotherapy with a BRAF inhibitor or combination therapy with BRAF and MEK inhibitors) between 1 July, 2012 and 1 June, 2019 were included. The ULN was defined at 250 U/L. Patients with prior systemic treatment for metastasized disease were excluded to avoid bias of ongoing activity of previous systemic agents.

### 4.3. Statistical Analysis

Time to next treatment (TTNT) and overall survival (OS) with corresponding two-sided 95% confidence intervals (CI) for medians were analyzed using the Kaplan–Meier method. For the overall study population, TTNT was determined from the start of targeted therapy to the start of subsequent systemic therapy, or death from any cause. Patients who were still on treatment were censored at time of analysis. OS was defined as the time from start of targeted therapy to the date of death from any cause. Patients alive at time of analysis were censored. Follow-up time was calculated from start date of targeted therapy using the inverse Kaplan–Meier method [22].

The main objective of the study was to investigate whether the response to targeted therapy and level of serum LDH at start of subsequent treatment with ICI affects survival. For this analysis, OS was defined from start of subsequent treatment with ICI to the date of death from any cause. Patients were stratified according to LDH at start of subsequent treatment with ICI (<ULN, >1 to <2× ULN, ≥2× ULN) and tumor response after treatment of targeted therapy according to Response Evaluation Criteria in Solid Tumors (RECIST). OS was compared between the subgroups using log-rank tests. Statistical significance was defined as a two-sided *p* value < 0.05. All statistical analyses were performed in PASW Statistics version 20 (SPSS Inc. Chicago, IL, USA).

## 5. Conclusions

This population-based study confirms the very poor prognosis of advanced melanoma patients with LDH ≥ 2× ULN. Moreover, our data suggest that switching to ICI upon response to targeted therapy with normalization of LDH may be a strategy to obtain long-term survival for these patients. Nevertheless, randomized trials are needed to assess the real benefit of sequential treatment of targeted therapy and ICI in patients with highly elevated serum LDH.

## Figures and Tables

**Table 1:** Patient and treatment characteristics of overall study population.

Variable	*n* = 360
	*n* (%)
Median age, years (IQR)	60 (50–68)
Age in categories	
<50	90 (25)
50–59	85 (23)
60–69	110 (31)
≥70	75 (21)
Gender	
Male	214 (60)
Female	146 (40)
ECOG PS	
0	75 (21)
1	110 (31)
≥2	136 (38)
Unknown	39 (10)
Median baseline LDH (IQR)	823 (625–1419)
No. of organ sites involved	
<3	60 (17)
≥3	261 (72)
Unknown	39 (11)
M stage	
M1a	6 (2)
M1b	3 (1)
M1c	349 (96)
Unknown	2 (1)
Brain metastases	
No	231 (64)
Asymptomatic	42 (12)
Symptomatic	63 (17)
Unknown	24 (7)
Type of targeted therapy	
BRAFi monotherapy	206 (76)
BRAFi + MEKi	154 (24)

IQR = interquartile range; ECOG PS = Eastern Cooperative Oncology Group performance status; BRAFi = BRAF inhibitor; MEKi = MEK inhibitor.

**Table 2:** Patient and treatment characteristics at start of subsequent treatment with ICI.

Variable	*n* = 113
	*n* (%)
Median age, years (min–max)	56 (47–67)
Age in categories	
<50	35 (31)
50–59	28 (25)
60–69	30 (26)
≥70	20 (18)
Gender	
Male	69 (61)
Female	44 (39)
Serum LDH	
<ULN	38 (34)
≥1 to <2× ULN	42 (37)
≥2× ULN	33 (29)
ECOG PS	
0	24 (21)
1	61 (54)
≥2	15 (13)
Unknown	13 (12)
No. of organ sites involved	
<3	17 (15)
≥3	84 (74)
Unknown	12 (11)
M stage	
M1a	0 (0)
M1b	0 (0)
M1c	113 (100)
Unknown	0 (0)
Brain metastases	
No	68 (60)
Asymptomatic	18 (16)
Symptomatic	19 (17)
Unknown	8 (7)
Type of prior targeted therapy	
BRAFi monotherapy	41 (36)
BRAFi + MEKi	72 (64)
Response on targeted therapy	
Partial response	27 (24)
Stable disease	7 (6)
Progressive disease	79 (70)
Type of subsequent ICI	
Ipilimumab	22 (19)
Nivolumab	16 (14)
Pembrolizumab	20 (18)
Ipilimumab and nivolumab	55 (49)

ECOG PS = Eastern Cooperative Oncology Group performance status; BRAFi = BRAF-inhibitor; MEKi = MEK inhibitor. LDH = lactate dehydrogenase, ULN = upper limit of normal; ICI = immune checkpoint inhibitors.

**Table 3:** Kaplan–Meier estimates of median overall survival, and 6-months and 1-year survival rates at start of subsequent treatment with ICI, according to serum LDH at start of subsequent treatment with ICI and tumor response after targeted therapy.

Serum LDH at Start ICI	Response on Targeted Therapy	Deaths/No. of Patients	Median OS	6 m Survival Rate	1 y Survival Rate
			(95% CI), m	(95% CI), %	(95% CI), %
<ULN					
	PR	7/16	24.7 (16.1–33.4)	85 (66–100)	73 (46–100)
	SD	5/6	7.0 (0–14.9)	63 (21–100)	21 (0–57)
	PD	14/16	4.4 (1.3–7.4)	36 (11–61)	22 (0–44)
≥1 to <2× ULN ^a^					
	PR	5/9	10.4 (0–22.5)	60 (24–96)	40 (0–80)
	PD	25/32	2.7 (1.9–3.5)	24 (8–40)	20 (5–35)
≥2× ULN ^b^					
	PD	29/31	1.1 (0.7–1.6)	17 (3–30)	8 (0–19)

LDH = lactate dehydrogenase, ULN = upper limit of normal, ICI = immune checkpoint inhibitors, OS = overall survival, m = months, NR = not reached, PR = partial response, SD = stable disease, PD = progressive disease. ^a^ Due to low numbers of patients with stable disease (*n* = 1) in this subgroup, these patients were excluded from analyses. ^b^ Due to low numbers of patients with partial response (*n* = 2) in this subgroup, these patients were excluded from analyses.

## Supplementary Materials

The following are available online at https://www.mdpi.com/2072-6694/11/12/1940/s1, Table S1: Patient and treatment characteristics at start of targeted therapy, according to subgroup of normalized LDH and PR (*n* = 16) and all other groups (*n* = 97) after targeted therapy.
