# Supplementary material for: Switching to Immune Checkpoint Inhibitors upon Response to Targeted Therapy; The Road to Long-Term Survival in Advanced Melanoma Patients with Highly Elevated Serum LDH?
Source: Cancers (Basel). 2019 Dec 4;11(12):1940. doi: 10.3390/cancers11121940 (PMC6966631; doi:10.3390/cancers11121940)
Supplement: Supplementary file 1 [file cancers-11-01940-s001.pdf]

Supplementary Materials: Switching to Immune Checkpoint Inhibitors upon Response to Targeted Therapy; The Road to Long-Term Survival in Advanced Melanoma Patients with Highly Elevated Serum LDH?

Maartje G Schouwenburg, Karijn P.M. Suijkerbuijk, Rutger H.T. Koornstra, Anouk Jochems, Michiel C.T. van Zeijl, Alfons J.M. van den Eertwegh, John B.A.G. Haanen, Maureen J.B. Aarts, Alexander C.J. van Akkooi, Franchette W.P.J. van den Berkmortel, Jan Willem B. de Groot, Geke A.P. Hospers, Ellen Kapiteijn, Wim H. Kruit, Djura Piersma, Rozemarijn S. van Rijn, Albert J. ten Tije, Gerard Vreugdenhil, Jacobus J.M. van der Hoeven and Michel W.J.M. Wouters

Table S1. Patient and treatment characteristics at start of targeted therapy, according to subgroup of normalized LDH and PR (*n* = 16) and all other groups (*n* = 97) after targeted therapy.

Variable	***n* = 16**	***n* = 97**	
	***n* (%)**	***n* (%)**	***P***
Median age, yrs (IQR)	60 (44–68)	56 (48–67)	0.29
Age in categories			
<50	6 (38)	29 (30)	
50–59	2 (12)	26 (27)	0.67
60–69	5 (31)	25 (26)	
≥70	3 (19)	17 (17)	
Gender			
Male	7 (44)	62 (64)	0.13
Female	9 (56)	35 (36)	
ECOG PS			
0	4 (25)	22 (23)	
1	6 (38)	40 (41)	0.98
≥2	5 (31)	28 (29)	
Unknown	1 (6)	7 (7)	
Median LDH value (IQR)	648 (554–1144)	788 (603–1273)	0.13
No. of organ sites involved			
<3	0 (0)	18 (19)	0.07
≥3	12 (75)	69 (71)	
Unknown	4 (25)	10 (10)	
M stage			
M1a	0(0)	0(0)	
M1b	0(0)	0(0)	
M1c	16 (100)	97 (100)	
Brain metastases			
No	8 (50)	72 (74)	
Asymptomatic	3 (19)	9 (9)	0.17
Symptomatic	2 (13)	10 (9)	
Unknown	3 (19)	6 (6)	

IQR = interquartile range; ECOG PS = Eastern Cooperative Oncology Group performance status; LDH=lactate dehydrogenase; [a] Categorical variables were compared using the chi-square test. Continuous variables were compared using the independent two-sample *t*-test.

© 2019 by the authors. Licensee MDPI, Basel, Switzerland. This article is an open access article distributed under the terms and conditions of the Creative Commons Attribution (CC BY) license (http://creativecommons.org/licenses/by/4.0/).
